# High-responsivity UV-Vis Photodetector Based on Transferable WS_2_ Film Deposited by Magnetron Sputtering

**DOI:** 10.1038/srep20343

**Published:** 2016-01-29

**Authors:** Longhui Zeng, Lili Tao, Chunyin Tang, Bo Zhou, Hui Long, Yang Chai, Shu Ping Lau, Yuen Hong Tsang

**Affiliations:** 1 Department of Applied Physics, The Hong Kong Polytechnic University, Hung Hom, Kowloon, Hong Kong

## Abstract

The two-dimensional layered semiconducting tungsten disulfide (WS_2_) film exhibits great promising prospects in the photoelectrical applications because of its unique photoelectrical conversion property. Herein, in this paper, we report the simple and scalable fabrication of homogeneous, large-size and transferable WS_2_ films with tens-of-nanometers thickness through magnetron sputtering and post annealing process. The produced WS_2_ films with low resistance (4.2 kΩ) are used to fabricate broadband sensitive photodetectors in the ultraviolet to visible region. The photodetectors exhibit excellent photoresponse properties, with a high responsivity of 53.3 A/W and a high detectivity of 1.22 × 10^11^ Jones at 365 nm. The strategy reported paves new way towards the large scale growth of transferable high quality, uniform WS_2_ films for various important applications including high performance photodetectors, solar cell, photoelectrochemical cell and so on.

The successful isolation of graphene from graphite has attracted extensive amounts of attention due to its attractive electrical and mechanical properties^1^,^2^, and its great potentials in advanced electronic and photonic applications^3^,^4^,^5^,^6^,^7^,^8^. However, the zero bandgap of graphene limits its application in optoelectronics. Recently, the emergence of two-dimensional (2D) graphene-like transition metal dichalcogenides (TMDs) have very well remedied the zero-bandgap disadvantage and attracted tremendous attention due to its semiconducting properties and potential for various optical, electrical and photoelectrical applications^9^,^10^,^11^,^12^. WS_2_ and MoS_2_ are the two most typical TMDs with layered structure constructed of S-W/Mo-S atomic tri-layer units^13^,^14^. Compared with MoS_2_, WS_2_ has higher thermal stability and wider operational temperature range^15^, possessing a controllable bandgap ranging from 1.4 to 2.1 eV depending on the proper layer structure^11^,^16^, leading to a broad UV to Visible (UV-Vis) absorption band. Furthermore, WS_2_ has been reported to have strong absorbance as high as 5~10% of incident sunlight in the thickness of ~1 nm, one order of magnitude higher than GaAs and Si^17^, showing great potential for photocatalysis, solar cells and high-responsivity UV-Vis photodetector applications.

Preparation of uniform large area WS_2_ thin film is the most fundamental step for various device fabrications. WS_2_ films can be prepared through top-down or bottom-up methods. Top-down method through mechanical or chemical exfoliation from the bulk crystals form have difficulty to control the size and thickness and obtain uniform WS_2_ films^18^,^19^,^20^. Among the bottom-up methods, chemical vapor deposition (CVD) can commonly produce large-area single-crystal 2D films, such as monolayer graphene^21^, however for TMDs (MoS_2_, WS_2_), there has been no reports on the preparation of wafer scale single-crystal films through CVD method yet, except for the preparation of micrometer sized triangular MoS_2_ and WS_2_^22^,^23^,^24^. As for the preparation of polycrystalline TMD films, the magnetron sputtering deposition method has proved to be better than the CVD method because of its simplicity, low cost, high production speed and scalability.

In this work, a facial and scalable magnetron sputtering method was employed to fabricate the transferable continuous centimeter sized polycrystalline WS_2_ film with thickness of ~25.2 nm, which is then used to produce high performance UV-Vis photodetectors, showing excellent photo-response properties from the ultraviolet to visible region (365–650 nm), such as high responsivity (53.3 A/W), high detectivity (up to ≈10^11^ Jones) at 365 nm. Therefore it is expected that this transferable high quality large-area WS_2_ films has great potential applications in not only photodetectors but also other photoelectrical functional devices.

## Results

**Figure 1:**
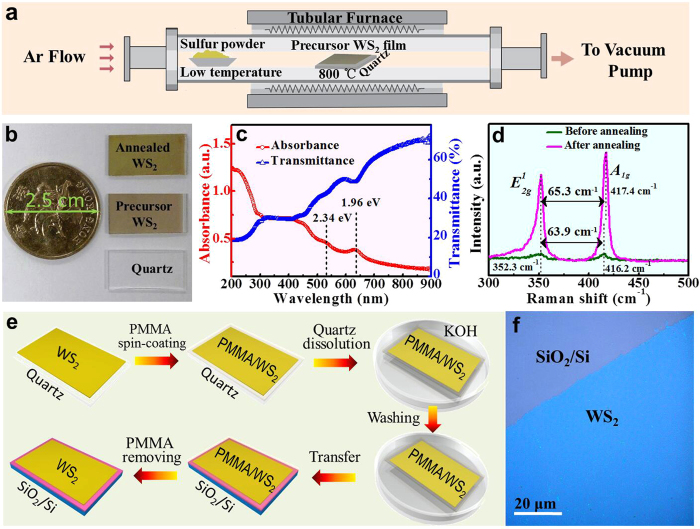
Preparation and transfer of WS_2_ film. (**a**) Schematic diagram of the annealing process for WS_2_ films. (**b**) A photograph of quartz and WS_2_ on quartz before and after annealing. (**c**) Measured UV-Vis absorption and transmission spectra of the annealed WS_2_ films. (**d**) Raman spectra of WS_2_ films before and after annealing. (**e**) Scheme of the transferring process of grown WS_2_ film from Quartz to SiO_2_/Si substrate. (**f**) Optical image of the transferred WS_2_ film on SiO_2_/Si substrate.

WS_2_ films on quartz substrate were produced by magnetron sputtering followed by post annealing. A WS_2_ precursor film was first deposited on clean quartz substrate through sputtering by using a sintered WS_2_ disk as the target. The post annealing process was then carried out to enhance the crystallinity of the WS_2_ film. Figure 1a shows the schematic diagram of the annealing process, the WS_2_ precursor film on quartz was loaded in a tubular reactor, and sulfur powder was placed upstream into the chamber. Sulfur was evaporated at 200 °C and dragged by Argon flow, and the center temperature of the tube furnace was set to 800 °C and kept for 2 hours, followed by cooling down to room temperature naturally to complete the annealing processes. Figure 1b shows the photographs of bare quartz, the WS_2_ precursor film and annealed film on the quartz substrates used for this experiment and their relative size as a one dollar coin from Hong Kong of 2.5 cm diameter. The gray sputtered WS_2_ precursor film shows a characteristic yellowish green color of few-layer WS_2_ crystal after annealing.

In Fig. 1c, the optical absorption and transmittance spectra of the annealed WS_2_ film are presented. Generally, the WS_2_ film possesses considerable photo-absorption covering the entire UV-Vis range of 200–900 nm, demonstrating the very broad ultraviolet to visible range photoelectrical response from the fabricated WS_2_ film based photodetector. Figure 1c records two characteristic absorption peaks at 633 nm (1.96 eV) and 530 nm (2.34 eV) arising from direct transition from valance band to conduction band at the K-point of the Brillouin zone, known as the A and B transitions, respectively. The two peaks formed due to the spin-orbital splitting of the valence band. In addition, the characteristic C peak centered at 455 nm (2.72 eV) is also observed^25^,^26^. As is well known that, the bandgap becomes smaller for the thicker WS_2_^16^, all the three peaks have exhibited a little red shift compared with the mono- and bi-layer WS_2_ reported, indicating the few-layer thickness of WS_2_ obtained here^26^.

The Raman spectra of theWS_2_ films before and after annealing under 488 nm excitation were obtained and presented in Fig. 1d. The two characteristic peaks corresponding to the E^1^_2g_ mode, in-plane vibration of tungsten and sulfur atoms, and A_1g_ modes, the out-of-plane vibrations of the sulfur atoms, are observed from the both two films^27^. The Raman peaks are identified at 352.3 and 417.4 cm^−1^ for annealed WS_2_ films, and 352.3 and 416.2 cm^−1^ for precursor WS_2_ films, very close to the reported result of few-layered WS_2_^10^, the frequency difference of the two Raman peaks of the annealed WS_2_ film is 65.1 cm^−1^, indicating the film is made up of a few layered WS_2_ nanostructure. The peak intensity of annealed WS_2_ film is much stronger compared with the pre-annealed WS_2_ film, which are contributed to the improvement of crystallinity resulting in strengthened lattice dynamics. Another unique feature observed in the Raman spectra is that the intensity ratio of A_1g_/E^1^_2g_ of the WS_2_ film increased from ~0.97 to ~1.24 after annealing, and the A_1g_ peak of the WS_2_ film slightly shifted to higher frequency while E^1^_2g_ peak remained the same after annealing, indicating A_1g_ mode which represents the out-of-plane vibrations of the sulfur atoms has become more sensitive to post annealing process.

Figure 1e shows that the WS_2_ films growth on the quartz can be transferred to other substrates such as SiO_2_/Si for device fabrication. First, PMMA was spin-coated on the WS_2_/quartz sample, followed by being immersed in potassium hydroxide (KOH) solution at 80 °C. KOH can etch the quartz substrate and then the PMMA/WS_2_ film will float on the solution surface. The floating PMMA/WS_2_ film was then washed with DI water for several times before being transferred to the new SiO_2_/Si substrate and dried. Then the PMMA was finally dissolved in acetone. The optical image of the transferred WS_2_ film on SiO_2_/Si shown in Fig. 1f indicates the WS_2_ film kept flat continuous after being transferred.

**Figure 2:**
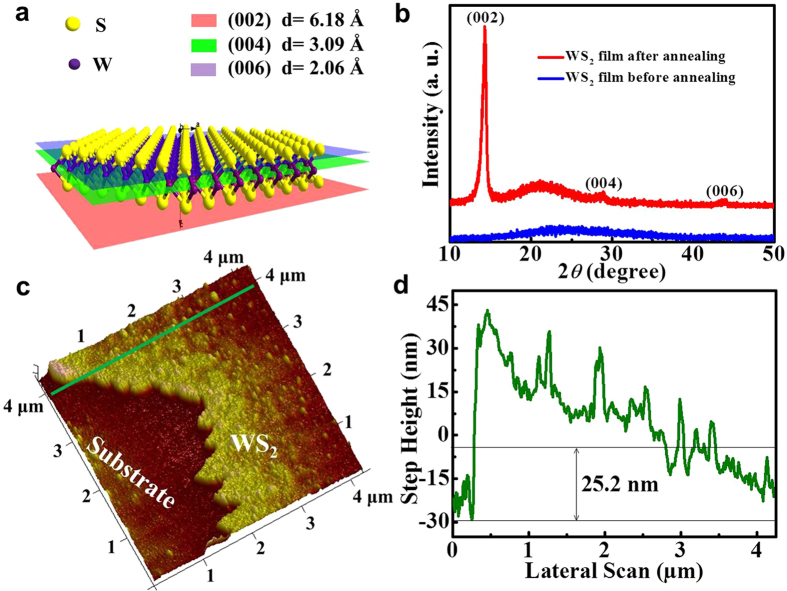
Characterization of WS_2_ film. (**a**) Three-dimensional atomic structure diagram of monolayer WS_2_. (**b**) XRD profile of WS_2_ films on the quartz before and after annealing. (**c**) Three dimensional AFM image of annealed WS_2_ film. (**d**) Height profile along the line marked in (**c**).

X-ray diffraction (XRD) method was carried out to investigate the crystalline structure and the composition of the WS_2_ films. It is well known that each single plane of WS_2_ comprises a tri-layer composed of a tungsten layer sandwiched between two sulfur layers as shown in Fig. 2a, with the (002), (004) and (006) crystal planes along the c direction being also presented in it. As observed from the measured XRD patterns shown in Fig. 2b, the XRD pattern of the precursor WS_2_ film shows no detectable diffraction peaks, indicating it is close to amorphous state. However, three diffraction peaks located at 14.31°, 29.02° and 43.74°, are observed from the annealed WS_2_ film, corresponding to the (002), (004) and (006) crystal planes of hexagonal WS_2_, respectively, confirming the well layer stacking along the c direction.

Figure 2c shows a three-dimensional atomic force microscope (AFM) surface topography scan of the annealed WS_2_ film, showing a little edge of the film, mainly because of the cluster effect at the edge during the sputtering and annealing process^9^. Figure 2d presents the section height profile of the WS_2_ film located at the marked position in Fig. 2c, confirming the thickness of the annealed WS_2_ film at about ~25.2 nm.

**Figure 3:**
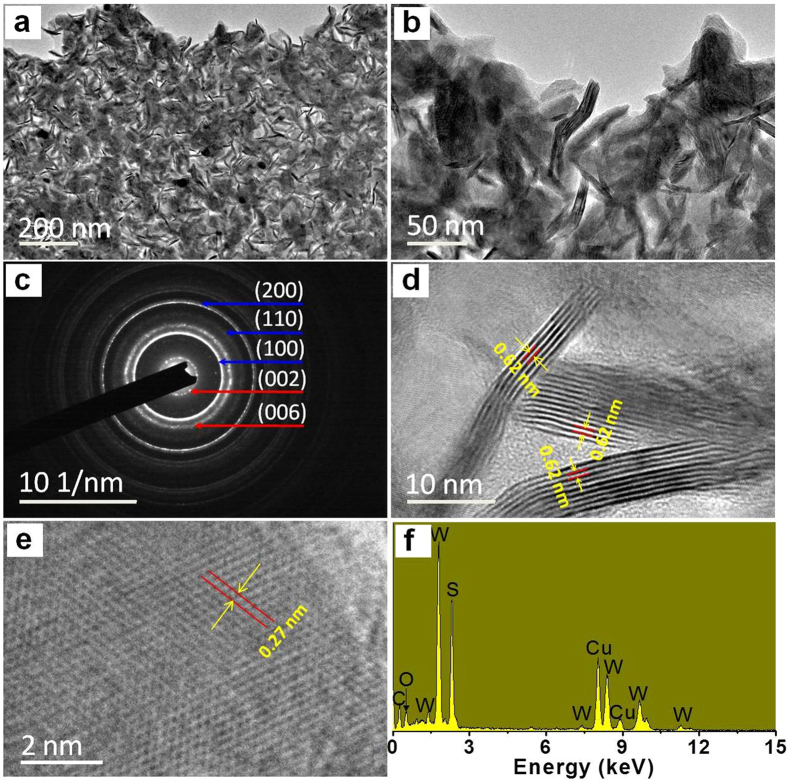
TEM image and analysis of WS_2_ film. (**a,b**) TEM images of transferred WS_2_ on the carbon-coated copper net. (**c**) The SAED pattern of the WS_2_. (**d**) HRTEM image of the vertically grown WS_2_ nanosheets. (**e**) HRTEM image of the horizontally grown WS_2_ nanosheets. (**f**) EDS spectrum of the sample.

Figure 3a,b shows the TEM images of WS_2_ at different magnifications, revealing that the WS_2_ film is composed of countless WS_2_ nanosheets, most of which are horizontal, and few of them are vertically grown. Figure 3c is the SAED pattern of the WS_2_ nanosheets, the diffraction rings from inside to outside are ascribed to the (002), (100), (006), (110), (200) planes, respectively, as marked in the figure^24^,^28^. Figure 3d gives the HRTEM image of the vertically grown WS_2_ nanosheets, showing the 0.62 nm interlayer distance of WS_2_, identical to the theoretical interlayer distance along the c-axis direction^29^. Figure 3e displays that the HRTEM image of the horizontally grown WS_2_, with 0.27 nm interplane spacing, matches well with the (110) planes, indicating a top view through the c-axis direction^30^. The EDS spectrum of the sample shown in Fig. 3f confirms the tungsten (W) and sulphur (S) elements are contained in the prepared sample, the existence of carbon (C), oxygen (O) and copper (Cu) are due to the carbon-coated copper substrate for TEM measurement.

## Discussion

**Figure 4:**
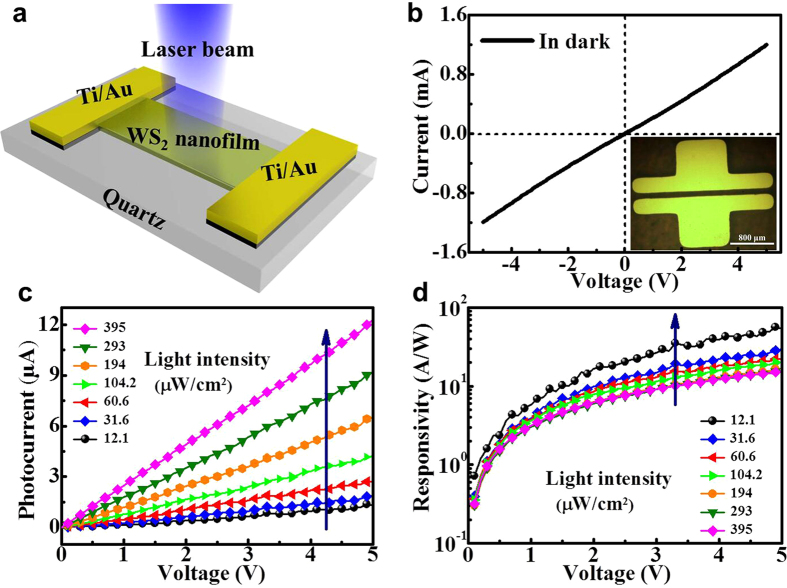
Device structure and photoresponse of photodetector. (**a**) Schematic representation of the photodetector consisting of a WS_2_ film and the Ti/Au contacts on quartz, and the laser sources were applied perpendicularly to the film. **(b**) Current-voltage plot obtained without illumination, the inset displays the microscope image of a pair of the Ti/Au electrodes. (**c**) I-V curves of the device obtained under different light intensities from 12.1 to 395 μW/cm^2^ at a wavelength of 365 nm. (**d**) The corresponding responsivity *vs.* voltage plots acquired under various light intensities.

Figure 4a schematically illustrates the fabricated photodetector based on the annealed WS_2_ film. The Ti (5 nm)/Au (50 nm) electrodes were deposited on top of the WS_2_ film by thermal evaporation method. A stainless steel shadow mask was used to pattern these electrode pads spaced 100 micrometers apart, and the Ti/Au contacts were captured by a CCD microscope and presented in the inset of the Fig. 4b, the photodetector was attached to a chip carrier fixture for electrical testing. Figure 4b shows the current-voltage curve of the annealed WS_2_ film measured in absence of light. The quasi-linear and symmetric for small bias voltages indicates ohmic contact between the WS_2_ channel and the Ti/Au electrodes. Then, the resistance at room temperature was calculated to be 4.2 kΩ according to the I-V curve shown in Fig. 4b, lower than other ones listed in Table 1, suggesting high quality, conductivity and uniformity of the as-prepared WS_2_ film.

To further reveal the photoelectrical response properties of the WS_2_ based photodetector, the bias voltage dependent photocurrent of the photodetector at 365 nm was measured and given in Fig. 4c, showing a series of I-V curves under increasing incident light intensity (from 12.1 to 395 μW/cm^2^), from which one can see the highly linear photocurrent of device depending on the bias voltage, indicating the ohmic-like contact between the WS_2_ channel and the Ti/Au electrodes. As seen from Fig. 4c,d both the photocurrent and responsivity increase with the applied voltage. Under higher bias voltage, the photo-generated electron-hole pairs can be more effectively separated and captured, with the increase in the carrier drift velocity and then the reduction in carrier transfer time *T*_*t*_ (defined as *T*_*t*_ = *l*^2^/*μV*, where *l* is the device channel length, *μ* is the carrier mobility and *V* is the bias voltage)^31^, finally resulting in the higher photocurrent and responsivity.

**Figure 5:**
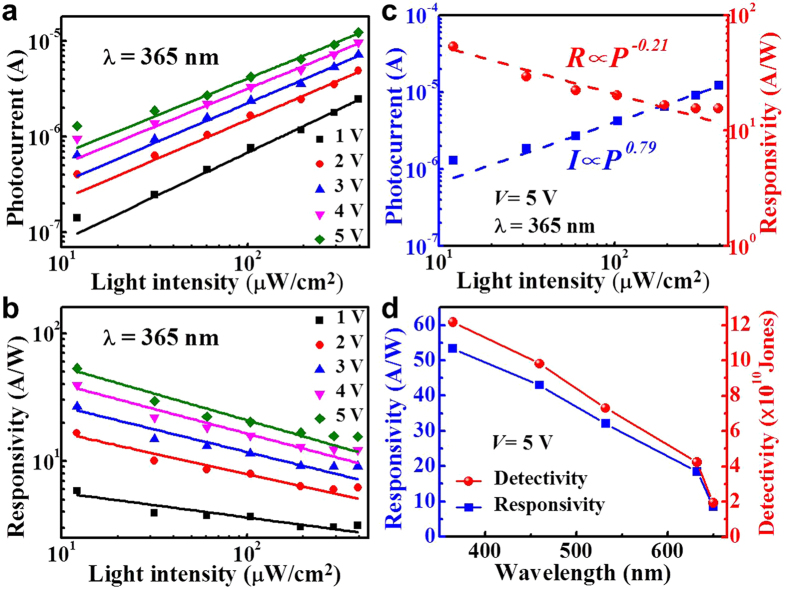
The performance of WS_2_ film as photodetector. (**a**) Photocurrent *vs.* light intensity and (**b**) Responsivity *vs.* light intensity plots acquired at different voltages varying from 1 to 5 V. (**c**) Photocurrent and responsivity as functions of light intensity at a 5 V bias voltage. The curves are fitted according to the power law *I*_ph_ = *AP*^α^. (**d**) Responsivity and photo-detectivity dependence on wavelength obtained at a 5 V bias voltage.

Figure 5a shows the photocurrent of the detector increases linearly with the incident excitation light intensity at the wavelength of 365 nm under various applied voltages. Under higher illumination intensity, more electro-hole pairs are generated in WS_2_, with the electrons and holes being directed into different electrodes guided by the applied electric field, resulting in the increase of the channel photocurrent. As shown in Fig. 5b, however, the responsivity of the photodetector reduced with increasing light intensity. This phenomenon should be attributed to the trap states containing defects and charged impurities within the WS_2_ film and at the interface between the quartz and WS_2_. Under low light intensity, the photo-generated electrons are mostly captured by the trap states and thus reduce the recombination of the electron-hole pairs. While under high illumination intensity, a relatively lower ratio of the photo-generated electrons will be captured because of the limited quantity of the trap states^31^. Thus, the device was more sensitive under lower light intensity^32^.

To analyze the quantitative dependence of the photocurrent on the illumination intensity, the photocurrent measured at *V* = 5 V as a function of light intensity is shown in Fig. 5c. The dependence of photocurrent on light intensity can be fitted by a simple power law: *I*_ph_ = *AP*^α^, where *I*_*ph*_ is the photocurrent, *A* is a scaling constant, *P* is the light power, and α is an exponent. The power equation fits (dashed lines) to the experimental data (solid squares) with an exponent (α = 0.79) between 0.5 and 1, indicating that the saturation is attributed to a kinetics of the photo-generated carriers that involves both recombination states and carrier-carrier interactions. The responsivity (*R*) is defined as the photocurrent generated per unit power of the incident light on the effective area and was obtained from the experimental data by using the formal *R* = *I*_ph_/*P*, and *a* quantitative fitting of the data yields a power law behavior *R = AP*^α−1^ with the fitting parameter α = 0.79 (*R*∝*P*^−0.21^) (see Fig. 5c).

The photocurrent responses of the device in the visible range are also measured and Fig. 5d depicts the calculated responsivity dependence on the wavelength, showing the responsivity of the WS_2_ photodetector increases at shorter spectral wavelengths. This is different from the common GaAs based photodetector of which the responsivity drops at reduced wavelength regions. The results also show the responsivity in the visible range are tens of A/W, much higher than the previously demonstrated records by using few-layered WS_2_ films based photodetectors (2.0~92μA/W)^10^, implying the capability of WS_2_ film based photodetector for conducting broadband photo-detection from the ultraviolet to visible region with promising high responsivity and its suitability for ultraviolet ultrasensitive photodetection. Moreover, to determine the sensibility of detecting a weak optical signal, the specific detectivity (D*) is measured and calculated. By assuming that shot noise from dark current constitutes a major contribution to the total noise, D^*^ can be expressed in the form of D^*^ = *A*^*1/2*^*R*/(2*qI*_*dark*_)^1^/2, where *R* is the responsivity, *A* is the effective area of the detector, *q* is the absolute value of electron charge, and *I*_dark_ is the dark current^33^. The D* is calculated to be 1.22 × 10^11^ Jones at a luminescent light intensity of 12.1 μW/cm^2^ at 365 nm and a bias voltage of 5 V. These results are superior or comparable to the previously reported photodetectors that are fabricated on the basis of 2D TMDs, as listed in Table 1. The wavelength dependent responsivity and detectivity of the device displayed in Fig. 5d clearly indicates better responsivity and detectivity at shorter wavelength regions (higher energy side).

In summary, we demonstrate a high performance UV-Vis broadband responsive photodetectors on the basis of low resistance large area WS_2_ film fabricated through magnetron sputtering and post-annealing process. The responsivity and detectivity of the photodetector can reach 53.3 A/W and 1.22 × 10^11^ Jones, respectively. Our results indicate that the magnetron sputtering method is a simple and scalable method for fabricating high quality WS_2_ films with diverse device applications, including photodetectors, solar cells, photoelectrochemical cells, phototransistor sensors and so on.

## Methods

### WS_2_ Film and Photodetector Fabrication

WS_2_ films on quartz substrate were produced by magnetron sputtering method followed by post annealing. The WS_2_ precursor film was first deposited on clean quartz substrate through sputtering by using sintered WS_2_ disks (Φ_2_*0.125 inch) bought from China New Metal Materials Technology Co, Ltd. as the target. The radio frequency (RF) power, argon gas pressure, and quartz substrate temperature were set to be 60 W, 50 Pa, and 200 °C, respectively, for the fabrication process. The deposition rate was about 5 nm min^−1^, leading to a 25.2 nm thick WS_2_ film after a deposition time of 5 min. The post annealing process was then carried out to enhance the crystallinity of the WS_2_ film. The WS_2_precursor film on quartz was loaded in a tubular reactor, and with sulfur powder (99.5% purity) being placed upstream in the chamber as shown in Fig. 1a. Sulfur was evaporated at 200 °C dragged by 100 sccm Argon flow. The center temperature of the tube furnace was set to 800 °C and kept stable for 2 hours, followed by being cooled down to room temperature naturally so as to complete the annealing processes. In fact, the post-annealing process can be done within the sputtering chamber by directly heating the substrate to high temperature under H_2_S atmosphere during the sputtering process^34^. The Ti/Au electrodes were deposited on top of the WS_2_ film by thermal evaporation using a stainless steel shadow mask to form channels (Width/Length = 2 mm/0.1 mm) for the electrical and photoelectrical measurements.

### Characterizations of Materials and Devices

UV-Vis absorption and transmission spectra were recorded on a SHIMADZU UV-2550 UV-Vis spectrophotometer. The Raman spectra measurements were carried out on a HR-800 Raman spectrometer with a 488 nm argon ion laser. The X-ray diffraction (XRD) pattern was recorded using a RigakuSmartLab X-ray diffractometer. The surface morphology and height information of samples were obtained by atomic force microscopy (AFM, VeecoNanoscope V).The morphology, crystal structure and chemical composition were investigated using a field emission transmission electron microscope (FETEM, JEOL Model JEM-2100F), equipped with an energy dispersive spectrometer (EDS). Additionally, to evaluate the photoresponse performance of the as-prepared WS_2_ film based photodetector, five laser sources operating at different wavelengths (365 nm, 460 nm, 532 nm, 632 nm and 650 nm) were used for the measurements. Electrical measurements were performed on a semiconductor characterization system (Keithley 2400-SCS).

## Additional Information

**How to cite this article**: Zeng, L. *et al.* High-responsivity UV-Vis Photodetector Based on Transferable WS_2_ Film Deposited by Magnetron Sputtering. *Sci. Rep.* **6**, 20343; doi: 10.1038/srep20343 (2016).

## Figures and Tables

**Table 1:** Summary of important device performance parameters of this work and other reported 2D layered transition metal dichalcogenides.

Materials	Fabrication Methods	Resistance	Responsivity^b^	Detectivity (Jones)^b^
Multilayer WS_2_^a^	Magnetron Sputtering	4.2 kΩ	53.3 A/W	~10^11^
Few-layer WS_2_^10^	CVD	~3 MΩ	92 μA/W	N/A
WS_2_ nanoflakes^15^	Mechanical Exfoliation	~4 MΩ	~20 A/W	N/A
Multilayer MoS_2_^12^	Magnetron Sputtering	N/A	~300 mA/W	~10^13^
Multilayer MoS_2_^35^	Mechanical Exfoliation	N/A	120 mA/W	~10^10^
Few-layer MoS_2_^9^	Thermolysis	N/A	700 mA/W	~10^10^
Graphene-MoS_2_ hybrid^36^	CVD	2.4 kΩ	62 A/W	N/A
Few-layer InSe^37^	Mechanical Exfoliation	~500 MΩ	~12 A/W	~10^11^

^a^This work.

^b^Maximum value reported on the experimental data.

## References

[b1] Geim A. K. Graphene: Status and Prospects. Science. 324, 1530–1534 (2009). 19541989 10.1126/science.1158877

[b2] Geim A. K. & Novoselov K. S. The Rise of Graphene. Nat. Mater. 6, 183–191 (2007). 17330084 10.1038/nmat1849

[b3] Bonaccorso F., Sun Z., Hasan T. & Ferrari A. C. Graphene Photonics and Optoelectronics. Nat. Photonics 4, 611–622 (2010).

[b4] Sun Z. *et al.* Infrared Photodetectors Based on CVD-Grown Graphene and PbS Quantum Dots with Ultrahigh Responsivity. Adv. Mater. 24, 5878–5883 (2012). 22936561 10.1002/adma.201202220

[b5] Wang Y., Qu Z., Liu J.& Tsang Y. H. Graphene Oxide Absorbers for Watt-Level High-Power Passive Mode-Locked Nd:GdVO_4_ Laser Operating at 1 Μm. J. Light. Technol. 30, 3259–3262 (2012).

[b6] Luo L. B. *et al.* Near-Infrared Light Photovoltaic Detector Based on GaAs Nanocone Array/monolayer Graphene Schottky Junction. Adv. Funct. Mater. 24, 2794–2800 (2014).

[b7] Zhao Y. *et al.* Mass Transport Mechanism of Cu Species at the Metal/Dielectric Interfaces with a Graphene Barrier. ACS Nano 8, 12601–12611(2014). 25423484 10.1021/nn5054987

[b8] Zhao J. Q. *et al.* L-Band Graphene-Oxide Mode-Locked Fiber Laser Delivering Bright and Dark Pulses. Laser Phys. 23, 075105 (2013).

[b9] Tsai D. S. *et al.* Few Layer MoS_2_ with Broadband High Photogain and Fast Optical Switching for Use in Harsh Environments. ACS Nano 7, 3905–3911(2013). 23590667 10.1021/nn305301b

[b10] Perea-Lopez N. *et al.* Photosensor Device Based on Few-Layered WS_2_ Films. Adv. Funct. Mater. 23, 5511–5517 (2013).

[b11] Georgiou T. *et al.* Vertical Field-Effect Transistor Based on Graphene-WS_2_ Heterostructures for Flexible and Transparent Electronics. Nat. Nanotechnol. 8, 100–103 (2013). 23263726 10.1038/nnano.2012.224

[b12] Wang L. *et al.* MoS_2_/Si Heterojunction with Vertically Standing Layered Structure for Ultrafast, High-Detectivity, Self-Driven Visible-Near Infrared Photodetectors. Adv. Funct. Mater. 25, 2910–2919 (2015).

[b13] Radisavljevic B., Radenovic A., Brivio J., Giacometti V. & Kis A. Single-Layer MoS_2_ Transistors. Nat. Nanotechnol. 6, 147–150 (2011). 21278752 10.1038/nnano.2010.279

[b14] Gong Y. *et al.* Vertical and in-Plane Heterostructures from WS_2_/MoS_2_ Monolayers. Nat. Mater. 13, 1135–1142 (2014). 25262094 10.1038/nmat4091

[b15] Huo N. *et al.* Photoresponsive and Gas Sensing Field-Effect Transistors Based on Multilayer WS_2_ Nanoflakes. Sci. Rep. 4, 5209 (2014). 24909387 10.1038/srep05209 PMC4048886

[b16] Kuc A., Zibouche N. & Heine T. Influence of Quantum Confinement on the Electronic Structure of the Transition Metal Sulfide TS_2_. Phys. Rev. B 83, 245213 (2011).

[b17] Bernardi M., Palummo M. & Grossman J. C. Photovoltaics Using Two-Dimensional Monolayer Materials Extraordinary Sunlight Absorption and one Nanometer Thick Photovoltaics Using Two-Dimensional Monolayer Materials. Nano Lett. 13, 3664–3670 (2013). 23750910 10.1021/nl401544y

[b18] Brivio J., Alexander D. T. L. & Kis A. Ripples and Layers in Ultrathin MoS_2_ Membranes. Nano Lett. 11, 5148–5153 (2011). 22010987 10.1021/nl2022288

[b19] Eda G., Yamaguchi H., Voiry D., Fujita T., Chen M. & Chhowalla M. Photoluminescence from Chemically Exfoliated MoS_2_. Nano Lett. 11, 5111–5116 (2011). 22035145 10.1021/nl201874w

[b20] Li H., Wu J., Yin Z. & Zhang H. Preparation and Applications of Mechanically Exfoliated Single-Layer and Multilayer MoS_2_ and WSe_2_ Nanosheets. Acc. Chem. Res. 47, 1067–1075 (2014). 24697842 10.1021/ar4002312

[b21] Li X. *et al.* Large-Area Synthesis of High-Quality and Uniform Graphene Films on Copper Foils. Science 324, 1312–1314 (2009). 19423775 10.1126/science.1171245

[b22] Schmidt H. *et al.* Transport Properties of Monolayer MoS_2_ Grown by Chemical Vapour Deposition. Nano Lett. 14, 1909–1913 (2014). 24640984 10.1021/nl4046922

[b23] Wang X. *et al.* Chemical Vapor Deposition Growth of Crystalline Monolayer MoSe_2_. ACS Nano 8, 5125–5131 (2014). 24680389 10.1021/nn501175k

[b24] Zhang Y. *et al.* Controlled growth of high-quality monolayer WS_2_ layers on sapphire and imaging its grain boundary. ACS Nano 7, 8963–8971 (2013). 24047054 10.1021/nn403454e

[b25] Zhao W. *et al.* Lattice Dynamics in Mono- and Few-Layer Sheets of WS_2_ and WSe_2_. Nanoscale 5, 9677–9683 (2013). 23999910 10.1039/c3nr03052k

[b26] Zhu B., Chen X. & Cui X. Exciton Binding Energy of Monolayer WS_2_. Sci. Rep. 5, 9218 (2014). 25783023 10.1038/srep09218 PMC4363839

[b27] Berkdemir A. *et al.* Identification of Individual and Few Layers of WS_2_ Using Raman Spectroscopy. Sci. Rep. 3, 1755 (2013).

[b28] Gutie H. R. *et al.* Extraordinary Room-Temperature Photoluminescence in Triangular WS_2_ Monolayers. Nano Lett. 13, 3447–3454 (2013). 23194096 10.1021/nl3026357

[b29] Late D. J., Liu B., Matte H. S. S. R., Rao C. N. R. & Dravid V. P. Rapid Characterization of Ultrathin Layers of Chalcogenides on SiO_2_/Si Substrates. Adv. Funct. Mater. 22, 1894–1905 (2012).

[b30] Du Y. *et al.* Improving the Anode Performance of WS_2_ through a Self-Assembled Double Carbon Coating. J. Phys. Chem. C 119, 15874–15881 (2015).

[b31] Lopez-Sanchez O., Lembke D., Kayci M., Radenovic A. & Kis A. Ultrasensitive Photodetectors Based on Monolayer MoS_2_. Nat. Nanotechnol. 8, 497–501 (2013). 23748194 10.1038/nnano.2013.100

[b32] Konstantatos G. *et al.* Ultrasensitive Solution-Cast Quantum Dot Photodetectors. Nature 442, 180–183 (2006). 16838017 10.1038/nature04855

[b33] Zeng L. *et al.* Bilayer Graphene Based Surface Passivation Enhanced Nano Structured Self-Powered near-Infrared Photodetector. Opt. Express 23, 4839–4846 (2015). 25836518 10.1364/OE.23.004839

[b34] Ellmer K. Preparation routes based on magnetron sputtering for tungsten disulfide (WS_2_) films for thin-film solar cells. phys. stat. sol. (b) 245, 1745–1760, (2008).

[b35] Choi W. *et al.* High-Detectivity Multilayer MoS_2_ Phototransistors with Spectral Response from Ultraviolet to Infrared. Adv. Mater. 24, 5832–5836 (2012). 22903762 10.1002/adma.201201909

[b36] Xu H. *et al.* High Responsivity and Gate Tunable Graphene-MoS_2_ Hybrid Phototransistor. Small 10, 2300–2306 (2014). 24664627 10.1002/smll.201303670

[b37] Tamalampudi S. R. *et al.* High Performance and Bendable Few-Layered InSe Photodetectors with Broad Spectral Response. Nano Lett. 14, 2800–2806 (2014). 24742243 10.1021/nl500817g

